# Non-invasive assessment and visualization of *Phytophthora cactorum* infection in strawberry crowns using quantitative magnetic resonance imaging

**DOI:** 10.1038/s41598-024-52520-7

**Published:** 2024-01-25

**Authors:** Teemu Valtteri Tuomainen, Anna Toljamo, Harri Kokko, Mikko Johannes Nissi

**Affiliations:** 1 Department of Technical Physics, University of Eastern Finland, Yliopistonranta 8, 70210 Kuopio, Finland; 2 Department of Environmental and Biological Sciences, University of Eastern Finland, Yliopistonranta 8, 70210 Kuopio, Finland

**Keywords:** Plant development, Biotic, Magnetic resonance imaging

## Abstract

*Phytophthora cactorum* is an oomycete species that causes enormous losses on horticultural crops, including strawberries. The purpose of this work was to investigate the alterations caused by *P. cactorum* inoculation in hydroponically grown strawberry plantlets (*Fragaria* × *ananassa* Duch.) using quantitative magnetic resonance imaging (qMRI). It was observed that with MRI, spatial and temporal progression of the infection could be observed in the crown using quantitative MR parameters, namely relaxation time maps. Relaxation times are numeric subject-specific properties that describe the MR signal behavior in an examined anatomical region. Elevated $T_{2}$ relaxation time values were observed inside the infected plant crowns with respect to the healthy references. The $T_{2}$ and $T_{2}^{*}$ values of healthy plants were small in the crown region and further diminished during the development of the plant. Furthermore, elevated $T_{1}$ relaxation time values were seen in regions where *P. cactorum* progression was observed in corresponding plant dissection photographs. Quantitative susceptibility maps (QSM) were calculated to estimate the local magnetic field inhomogeneities. The QSM suggests magnetic susceptibility differences near the center of the pith. This study provides novel non-invasive information on the structure and development of strawberry plants and the effects caused by the *P. cactorum* infection.

## Introduction

Garden strawberry (*Fragaria* × *ananassa* Duch.) is a hybrid species in the *Rosaceae* family with an estimated annual production of nearly 9 million tonnes^1^ of edible accessory fruit worldwide*.* Although a significant agricultural product and commodity, their growth and development can be severely hindered by a soil- and water-borne pathogen, *Phytophthora cactorum,* an oomycete species. Oomycetes are eukaryotic organisms, many of which are pathogenic to plants^2^. From these, *P. cactorum* can cause infections in over 200 different plants, including strawberries^3,4^. *P. cactorum* can survive for long periods in the soil as oospores^3^, and can thus, cause significant and continuous problems for horticulture.

**Figure 1:**
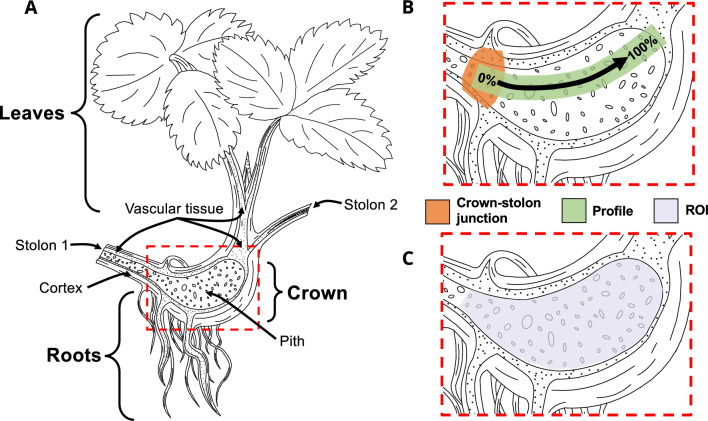
Simplified schematic illustration of strawberry plant structures and an example of the profile selection. (**A**) Simplified illustration of *Fragaria* × *ananassa* structures, namely leaves, crown and roots to assist in the interpretation of the MR images and quantitative data. Stolon 1 is connected to the mother plant and Stolon 2 to the daughter plant. (**B**) Example selection used for presenting average relaxation time profile data. The arrow indicates the direction and increase in distance from the crown-stolon junction in the profiles in Fig. 6. The presented approximate crown-stolon junction was used as a starting point for the profile. (**C**) Example of a region-of-interest (ROI) section for volumetric analysis of the pith region.

The anatomy of strawberry plants as well as growth and structural development have been previously investigated and described in several studies^5–8^. The main structures of a strawberry plant include the crown, from which adventitious roots as well as the additional stems and shoots originate (Fig. 1A). Although new strawberry plantlets can develop from the achenes on the surface of the accessory fruit, the typical reproduction occurs through asexual cloning of the mother plant by generating structures known as stolons originating from the crown. These elongated stems propagate ageotropically above ground and generate new plants at their nodes with an eventually self-sustaining structure, including crown, roots, stems, and leaves. The adventitious roots originate from the crown base, or as referred here, the crown-stolon junction, i.e. the intersection between the newly generated daughter plant and the stolon originating from the mother plant.

The strawberry crown rot infection caused by *P. cactorum* is often initiated by zoospores through the possible cut off stolon (stump) or from other wounds, such as rhizome injuries^3^. In the field, the infection is typically first observed as sudden wilting of the leaves. At this stage the disease has already severely progressed, and internal symptoms are clearly visible. Freshly invaded crown tissue has water-soaked appearance and light brown discoloration. As the infection and necrosis progress, the color turns darker, and the decay of the vascular system eventually causes the wilting of the plant. Sometimes, the plant may recover from *P. cactorum* attack, but usually normal growth and development remains hindered. Non-invasive methods capable of visualizing internal structures could be used to provide insight on disease progression and plant development at various time points.

At molecular level, *P. cactorum* expresses hundreds of genes encoding degradative enzymes to break down plant cell wall structures^9^. Alterations in the integrity of cell membranes are also likely to occur. The accumulation of lysophospholipids and free fatty acids, the hydrolysis products of phospholipids, is observed in the crown tissue of *Fragaria* species when plants are challenged with a virulent isolate of *P. cactorum*^10^. Lysophospholipids may act as signaling molecules in plant defense^11^. However, at high concentrations, they may disrupt membrane structures and evoke cell death promoting processes in plant tissues. As the *P. cactorum* infection involves elements that may affect the integrity of plant cell structures and membranes and thereby could alter the movement of water, solutes, and metabolites, magnetic resonance imaging (MRI) can be seen as a relevant tool for monitoring such activity.

Magnetic resonance imaging (MRI) is the spatially encoded extension of a phenomenon termed nuclear magnetic resonance (NMR). The application of NMR and MRI in various fields of plant science^12,13^ have been previously covered in literature. An important property in MRI is the capability to produce a variety of different image weightings and contrasts dependent on the utilized pulse sequence, imaging parameters and inherent sample properties, i.e., the molecular environment as well as the distribution and concentration of the measurable nuclei, most typically the hydrogen protons (^1^H nuclei) of water molecules. Furthermore, beyond these qualitative (anatomical) MR images, quantitative parameters, such as relaxation times can be measured. $T_{1}$ relaxation time describes the rate at which the excited MR signal recovers and can be re-excited and measured again, while $T_{2}$ and $T_{2}^{*}$ describe the rate at which the measurable signal diminishes^14^. Broadly, the more solid a region is, or the more interfaces of different materials a region has, the shorter $T_{2}$ and $T_{2}^{*}$ relaxation times are observed. For $T_{1}$ relaxation time, such generalization cannot be made, as both fast and slow molecular motion can cause an increase in the $T_{1}$ relaxation time value, depending on the frequency of the molecular motion with respect to the resonance frequency of the MRI device. In practice, relaxation times can be estimated pixel- or voxel-wise from a set of images obtained by varying an MR imaging parameter sensitive to the respective relaxation process. Besides relaxation times, relative magnetic susceptibility within the sample can be measured. Minute variations in the local field caused by polarized molecules can affect the measured signal. These variations can be due to accumulation of materials, such as air, minerals or various elements that are more dia-, para- or ferromagnetic than the surrounding bulk of the sample. Method known as quantitative susceptibility mapping (QSM)^15,16^ can be used to probe such properties of the sample.

NMR and MRI investigations regarding plant biology have been on occasion established with a variety of studies on fruits, seeds, shoot and stems as well as root and soil interactions^17–20^. Particularly in the case of *Fragaria* species, MRI has been utilized to qualitatively interpret the structure of fruits and flowers^21–24^ as well as freezing injuries in the crown^25^. Thus, while not entirely missing from the literature, only a few MRI investigations on strawberry species can be found, and most of them focus on the accessory fruit over the vegetative structures. Generally, the disease spread and progression in plants have been occasionally investigated with MRI. For example, the pathogenic processes in grapevine shoots^26^ have been previously investigated with qualitative MRI. In the case of strawberry, MRI of the interaction between the accessory fruit and progression of certain pathogens (*Botrytis cinerea, Colletotrichum acutatum,* and *Phytophthora cactorum*) have been briefly presented^27^. The previous study showed that a change in $T_{1}$ relaxation time could be attributed to the pathogen progression with clear differences between the healthy accessory fruit tissue, the leading edge of the disease, and the inactive regions caused by the pathogen. The knowledge of *Phytophthora cactorum* progression in plants is limited, as are the methods for investigating the internal disease progression in the same individual plant. Since MRI represents one of the few methods that allow such non-invasive investigations of plants, the purpose of this study was to evaluate the potential of using MR imaging for assessing the infection caused by *P. cactorum* in developing *Fragaria* × *ananassa* crowns, longitudinally, and compare the findings to those in intact plantlets.

## Results

In this work, the progression of crown rot disease caused by *P. cactorum*, was investigated with quantitative magnetic resonance imaging (qMRI) in the crown and surrounding structures of strawberry plantlets (Fig. 1A). This was accomplished by examining the development of healthy and inoculated plantlets ($N$ = 4 for both) as a function of time for a total of two weeks at three time points (designated as weeks 0, 1 and 2). For some investigated plantlets, the stolon cut-off was kept above the water level. This did not seem to affect the outcome of inoculation as after week 2, all inoculated plantlets had clear visual symptoms of a progressed infection.

**Figure 2:**
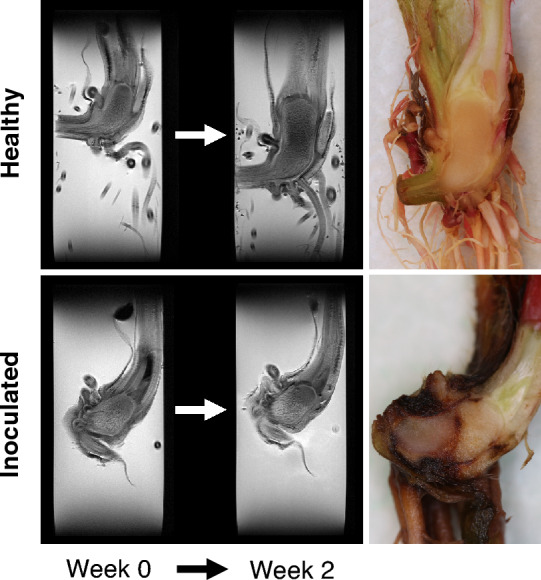
Raw MR images from MEMS data. MR images obtained with multi echo multi slice (MEMS) pulse sequence utilizing the shortest echo time (TE = 9.98 ms) of healthy and inoculated plantlets at different time points. Observe the free water surrounding the plantlets and the limited RF coil range near the upper and lower bounds of the image. The representative examples here are #1 from healthy and #2 from inoculated plantlets presented in Supplementary Fig. S1 online.

**Figure 3:**
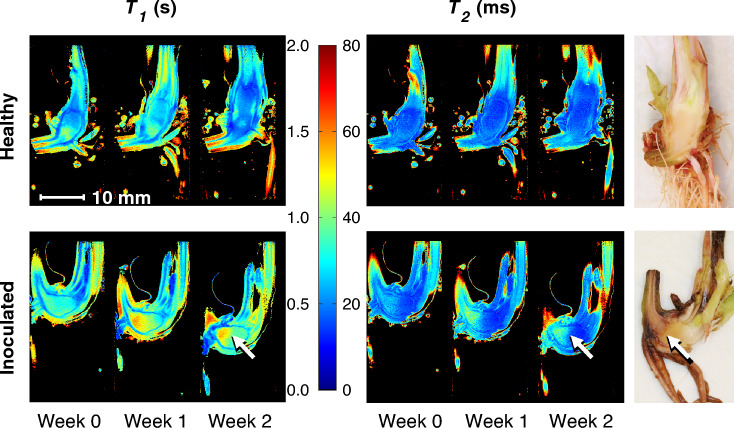
Examples of relaxation time maps of healthy and inoculated plants during development. Quantitative $T_{1}$ and $T_{2}$ relaxation time maps of healthy and inoculated strawberry crowns during a two-week development period after inoculation. $T_{1}$ and $T_{2}$ relaxation time maps are calculated from fast spin echo multi slice—inversion recovery (FSEMS-IR) and multi echo multi slice (MEMS) pulse sequence MRI data, respectively. Differences in the visibility of the stolon and root structures between time points is due to slightly different MR image slice positions. The representative examples here are #4 from healthy and #1 from inoculated plants presented in Supplementary Fig. S1 online. Relaxation time data from surrounding water have been omitted (black). The white arrow indicates the observed progressing edge of the infection in the dissection photograph as well as the same position at the relaxation time maps. The color gradient represents the relaxation times for each region, from relatively low relaxation time values (blue) to relatively high relaxation time values (red), and relaxation time values in-between (cyan, green, yellow and orange).

**Figure 4:**
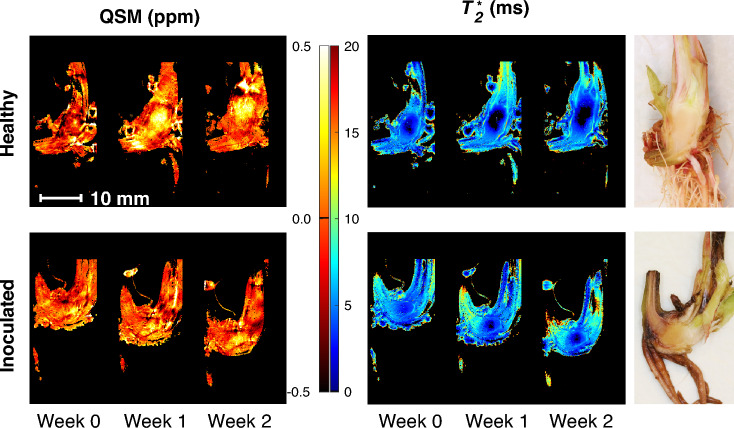
Example of QSM and $T_{2}^{*}$ relaxation time maps. Quantitative $T_{2}^{*}$ map and quantitative susceptibility map (QSM) of healthy and inoculated strawberry crowns during the two-week development period after inoculation. Mean of 10 consecutive slices from $T_{2}^{*}$ relaxation time maps and quantitative susceptibility maps (QSM) are presented. Averaging effectively provides the same slice thickness as obtained with $T_{1}$ fast spin echo multi slice—inversion recovery (FSEMS-IR) and $T_{2}$ multi echo multi slice (MEMS) data sets. Differences in the visibility of the stolon and root structures between time points are due to slightly different MR image slice positions. For comparison, similar positions with respect to the relaxation time maps in Fig. 3 were selected for these maps. The representative examples here are #4 from healthy and #1 from inoculated plantlets presented in Supplementary Fig. S1 online. Relaxation data from surrounding water have been omitted (black). For Fig. 4a, the color gradient represents magnetic susceptibilities from negative diamagnetic susceptibility (dark red) to positive paramagnetic susceptibility (light yellow), with zero susceptibilities in-between (orange). For Fig. 4b, the color gradient represents the relaxation times for each region, from relatively low relaxation time values (blue) to relatively high relaxation time values (red), and relaxation time values in-between (cyan, green, yellow and orange).

**Figure 5:**
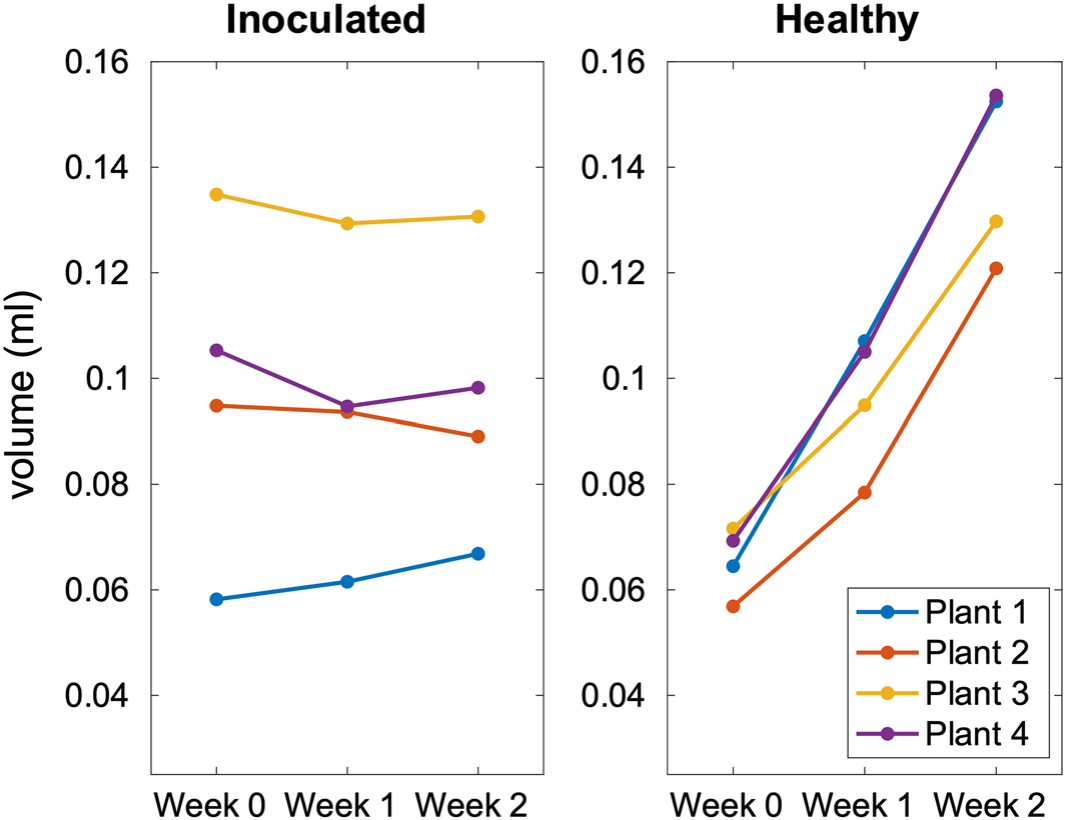
Volume of the crown pith. Manually segmented approximate crown pith volumes from MGE data (shortest TE) for healthy and inoculated plantlets.

Example MR image slices using multi echo multi slice (MEMS) pulse sequence (TE = 9.98 ms) are presented in Fig. 2 for healthy and inoculated plantlets submerged in water. For the healthy individual, growth is observable between the time points. The finite range of the transceiver RF coil is also observable, as signal intensity is reduced at the upper and lower parts of the images due to a large field of view (FOV). This is especially seen in free water surrounding the plantlets (Fig. 2). As the sensitivity of the transceiver RF coil decreases so does the effective flip angle, causing the signal decrease. From the measured MRI data, relaxation time maps ($T_{1}$, $T_{2}$ and $T_{2}^{*}$) were calculated for each time point. An example of calculated relaxation time maps ($T_{1}$ and $T_{2}$) of healthy and *P. cactorum* inoculated plants as well as a reference photograph of the corresponding plants dissected at the 2-week time point is presented in Fig. 3. In the relaxation time maps of the inoculated plants, it can be observed that the $T_{2}$ relaxation times are higher near the crown-stolon junction with an increase as a function of time. For $T_{2}^{*}$ relaxation times, higher values were seen near the crown-stolon junction, as in $T_{2}$ values (Fig. 4). Furthermore, for the $T_{2}^{*}$ relaxation time maps, a loss of signal near the center of the crown pith is observable in the healthy example. This is further exemplified by the quantitative susceptibility data, where higher positive susceptibility is observable in the healthy plant (Fig. 4). Volumetric analysis of the crown piths was conducted from the MGE data (for ROI selection please see Fig. 1C). Especially for the inoculated plants, the starting size of the plants at week 0 varied based on the volumetric analysis (Fig. 5).

**Figure 6:**
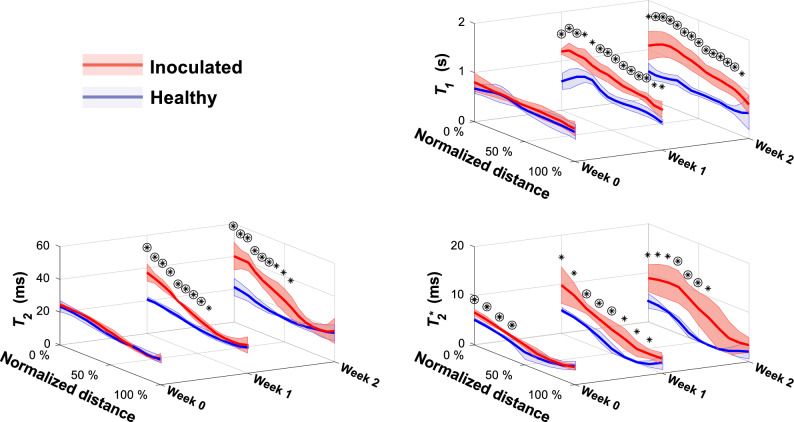
Average relaxation time profiles across the healthy and inoculated crown piths at different time points. Point-wise average profiles from $T_{1}$, $T_{2}$ and $T_{2}^{*}$ data (FSEMS-IR, MEMS and MGE pulse sequences) of 4 plantlets for both healthy and inoculated plants at all time points (week 0, 1 and 2). The normalized distance is illustrated in Fig. 1B and indicates the percentage from the crown-stolon junction. The circled asterisks above the profiles indicate highly statistically significant difference ($p$ < 0.01) and asterisks statistically significant difference ($p$ < 0.05).

**Figure 7:**
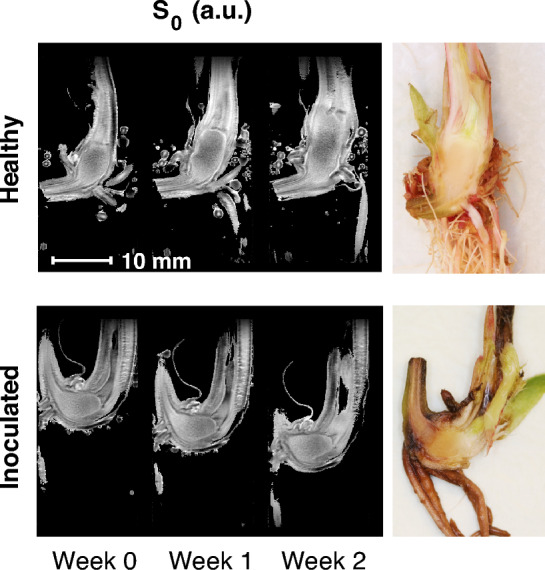
Example of proton density maps. Normalized average proton density $S_{0}$ maps of healthy and inoculated strawberry crowns during the two-week development period after inoculation. Average proton densities were calculated from $S_{0}$ maps obtained together with $T_{1}$ and $T_{2}$ relaxation time maps. Differences in the visibility of the stolon and root structures between time points are due to slightly different MR image slice positions. The representative examples here are #4 from healthy and #1 from inoculated plantlets presented in Supplementary Fig. S1 online. Values are presented in arbitrary units (a.u.) and the values from surrounding water have been omitted (black).

**Figure 8:**
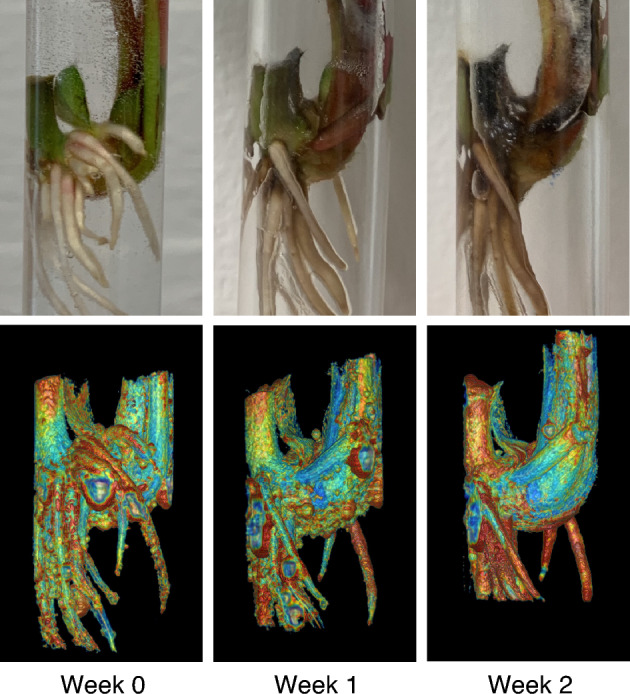
Development of an inoculated plantlet. External symptoms of the *Phytophthora cactorum* progression in a strawberry plantlet (#1) at the three time points (week 0, 1 and 2) together with 3-D pseudo-color volume renderings from $T_{2}^{*}$ relaxation time maps calculated from MGE data. Air bubbles are visible on the surface of the volume rendered plantlet. $T_{2}^{*}$ relaxation times from the surrounding water have been omitted. A rotating example video of dissected healthy and inoculated plantlets at different timepoints can be found in the online supplementary material (Supplementary Video S1 online). Color is used to illustrate the $T_{2}^{*}$ relaxation time values for different regions from relatively low relaxation time values (blue) to relatively high relaxation time values (red), and relaxation time values in-between (cyan, green, yellow and orange).

To analyze the entire relaxation time data set, regional relaxation time profiles were calculated for the pith of the crown, extending from the crown-stolon junction to the vascular tissue at the opposite end of the crown, illustrated in Fig. 1B. For both batches, arithmetic means and standard deviations of each time point were then investigated (Fig. 6). From here, it can be observed that the mean $T_{2}$ values near the crown-stolon junction have increased on weeks 1 and 2 for the inoculated plants, whereas the starting point on week 0 is nearly identical for healthy and inoculated plants. While $T_{2}$ exhibits spatial dependence within the pith of the crown, $T_{1}$ can be seen to increase, on average, along almost the entire profile on weeks 1 and 2 (Fig. 6). In the proton density maps $S_{0}$, calculated together with the $T_{1}$ and $T_{2}$ relaxation time maps, no significant visual differences are obvious in the crown pith region. A slight increase relative to the pith can be appreciated in the vascular tissue of the healthy plants, suggesting higher water content in these regions in the healthy plants as compared to those in the inoculated plants (Fig. 7). Three-dimensional pseudo-color volume renderings of an inoculated plant generated from $T_{2}^{*}$ data demonstrate the outer structures at different time points (Fig. 8). As can be seen from the 3-D volume renderings and photographs, the development has halted, and secondary root formation is non-existent; further 3-D visualizations of the intact and inoculated plants at different time points are available in the supplementary information as a video file. In the photographs (Fig. 8), darkening of the stolon stump is visible as a function of time. Similarly, clear differences in the photographs of the plants at week 2 can be observed between the healthy and the inoculated plants (see Supplementary Fig. S1 online).

For $T_{2}^{*}$ relaxation time maps obtained from the three-dimensional MGE data, mean of 10 consecutive slices was calculated to obtain a slice thickness of 1 mm, comparable to those of the examined $T_{1}$ and $T_{2}$ maps. From these $T_{2}^{*}$ maps, mean profiles were calculated (Fig. 6). Besides $T_{2}^{*}$ mapping, the MGE data was used for quantitative susceptibility mapping (QSM) to obtain information on the magnetic susceptibility of various plant regions. In the QSM data (Fig. 4), paramagnetic susceptibility (${\upchi }$ > 0 ppm) values were observed in the middle of the crown pith with higher values in the healthy plants and during later stages of plant development.

## Discussion

Quantitative magnetic resonance imaging was utilized to longitudinally investigate the changes in the crown of *P. cactorum* inoculated strawberry plantlets in hydroponic cultivation. It was observed that the obtained relaxation times were altered in the crown structures as a function of time for the inoculated plants and were significantly different with regards to the healthy plants used for reference. Moreover, the volumetric analysis of the crown piths clearly revealed that the development of the inoculated plants was halted, whereas the healthy subjects continued their natural growth (Fig. 5). While a size disparity was more obvious in the inoculated plants it does not seem to translate to the observed relaxation times or changes therein; relaxation time differences were observable irrespective of the starting volumetric size of the pith. After the halted development, the biological properties were severely altered, as revealed by the changes in the calculated relaxation times and dissection photographs.

The relaxation times describe the signal behavior in each pixel and together with imaging parameters are crucial for image formation as well as quality. Thereby, the findings presented here may be used to guide the selection of appropriate imaging parameters in future studies. $T_{2}$ relaxation time constant represents the NMR signal decay for a specific region or structure. Broadly, longer $T_{2}$ values are typically associated with high molecular tumbling rate, e.g. in liquid (free) water, whereas short and ultrashort $T_{2}$ are present in solid-like tissues with low tumbling rate. The calculated $T_{2}$ relaxation times in the strawberry crown near the crown-stolon junction were higher in the *P. cactorum* inoculated plants and were observed to increase between the measured time points. This could be associated with a breakdown of plant tissue, such as disintegration of cell walls and disruption of membranes, and concomitant increase in free water content. The most noticeable and prominent difference was between week 0 and week 1 in the pith near the crown-stolon junction (0–25% in Fig. 6, using a profile selection illustrated in Fig. 1B). As the adventitious roots mainly emerge from the approximate location of the crown-stolon junction, it may be that the *P. cactorum* is introduced to the crown region via this route.

For the plants inoculated with *P. cactorum*, both the $T_{1}$ and $T_{2}$/$T_{2}^{*}$ maps exhibit temporal alteration near the apparent progressing edge of the infection (Figs. 3, 4). $T_{1}$ is a region and substance specific parameter that describes NMR signal regeneration after excitation and decay. For healthy plants, $T_{1}$ appears to be spatially dependent, although in a different manner than for the inoculated plants, as a noticeable increase in $T_{1}$ relaxation time surrounding the pith of the crown is observed, possibly as a narrow band (Fig. 3). Previously it has been described, that pathogen (including *P. cactorum*) infected strawberry accessory fruit exhibit changes in the $T_{1}$ relaxation^27^. Unlike $T_{2}$ or $T_{2}^{*}$ values, $T_{1}$ relaxation times cannot be linearly attributed to the molecular motion and tumbling rate of the measured nuclei as both high and low tumbling rates can cause an increase in $T_{1}$. Thus, no clear description of the occurring phenomenon can be made from the behavior of $T_{1}$ relaxation alone. However, together with $T_{2}$ these can be used to describe sample properties and changes therein. In a previous study, an increase in $T_{2}$ has been observed during a fungal infection (*Botrytis cinerea* Pers.) in strawberry (accessory) fruit^28^, albeit only from qualitative $T_{2}$-weighted MR images.

The deterioration of cell walls and /or membranes may cause changes in $T_{2}$ relaxation times as the distribution and amount of free water changes. In the aforementioned strawberry study, *B. cinerea* damaged the cell walls of fruit parenchymal cells causing the leakage of cell fluids into the intercellular spaces and thereby the increase of $T_{2}$ values in the infected tissue^27^. In stored apples, internal browning regions exhibited higher average $T_{2}$ values compared to healthy tissue and a clear border adjacent to the healthy and browned area in MR images^29^, similar to that of the present study (Fig. 3). The observed changes in apples were explained to be related to partial destruction of cellular structures and higher free water content in the browning region. Interestingly, a virulent *P. cactorum* isolate has been previously observed to induce break down of membrane phospholipids to lysophospholipids and fatty acids in the strawberry crown tissue^10^. While these molecules may play roles in defense signaling, at higher concentrations they are capable of disrupting membrane structures and causing alterations to membrane permeability^11^. In addition, *P. cactorum* is known to express a plethora of degradative enzymes targeted against cell wall structures^9^. As water associated to cell walls is known to exhibit shorter $T_{2}$ values than free water^30^, the partial break-down of plant cell wall components may also contribute to changes in water distribution and increased $T_{2}$ values. Studies of metabolic changes in strawberry in response to a *P. cactorum* infection^10^ could be combined with MRI investigations in future.

Although not necessarily the only mechanism contributing to the signal increase and increase in $T_{2}$ relaxation times, cell deterioration could be a considerable component for the observed behavior. In general, however, other explanations for increased $T_{2}$ also exist, such as changes in metabolite contents. For example, Hajjar et al*.* investigated internal defects of potato tubers at 1.5 T and linked together increased $T_{2}$ and the absence of starch in the rust spot regions^31^. There, bi-exponential $T_{2}$ relaxation times were calculated to investigate the microstructural differences between rust spots and healthy tissue. They observed higher $T_{2}$ relaxation time values for the defective tissues than for an adjacent perimedullary region for both $T_{2}$ components^31^: for healthy tissues, the two $T_{2}$ components were approximately 70 ms and 220 ms. Conversely, the $T_{2}$ components for the rust spots were 137 ms and 485 ms and decreased to 107 ms and 398 ms during an 11-week period. Still, as with the present study, noticeably higher $T_{2}$ values are observable in the affected tissue values when compared to the healthy tissues. Although the numerical values are not directly comparable, the observed trend is similar. Hajjar et al*.* presented two potential hypotheses for observed differences in $T_{2}$: deterioration of membranes and increased mobility of water molecules, or lower starch content in rust spot regions. Of these, the lack of starch hypothesis was more supported by staining and microscopy findings. In biotic stress, however, the starch is usually accumulated rather than degraded^32^ and therefore, the effect of crown starch status on $T_{2}$ in our study is questionable. Furthermore, $T_{2}$ is also affected by static inhomogeneities in the magnetic field via diffusion and chemical exchange. As the spins move in inhomogeneous magnetic fields, such as those arising due to interfaces of materials with different magnetic susceptibilities during a finite measurement time, the spins may not be optimally refocused, and thus, part of the signal is lost. This phenomenon is observable in the $T_{2}$, albeit more in the measurements wherein $T_{2}^{*}$ relaxation is the mainly observed effect.

For three-dimensional visualization of $T_{2}^{*}$ relaxation times and plant structures, MGE data was collected. Similar patterns of increase near the crown-stolon junction were observed in the calculated $T_{2}^{*}$ relaxation time maps as with the $T_{2}$ relaxation times. However faster signal decay (lower numeric values) is observable in $T_{2}^{*}$ than in the calculated $T_{2}$ relaxation times. $T_{2}^{*}$ relaxation time is defined similarly to that of $T_{2}$ with the exception that a faster signal decay due to destruction of phase between individual nuclear spins is present due to minute variations in the local magnetic susceptibility. This can reduce $T_{2}^{*}$ with respect to $T_{2}$. To account for voxel and pixel dimension differences between MGE and MEMS pulse sequences, an arithmetic mean of 10 consecutive slices from $T_{2}^{*}$ maps was calculated (Fig. 4). Thus, $T_{2}^{*}$ maps more comparable to other relaxation time maps could be obtained. Interestingly the signal intensities in the measured MGE dataset were low near the center of the pith of the crown, especially for the healthy subjects. This is also apparent in the calculated $T_{2}^{*}$ maps with such low intensities masked out (Fig. 4). Similar, albeit smaller signal void can be seen in the inoculated plant as well (Fig. 4). As similar property is not observable in the $T_{2}$ maps (Fig. 3) quantitative susceptibility mapping was utilized to investigate the possible differences caused by changes in the local magnetic field in the pith.

Besides $T_{2}^{*}$ relaxation time maps, quantitative susceptibility maps (QSM) were calculated from the MGE data. QSM represents the magnetic susceptibility values for each pixel and region within a measured MR image and can be used to interpret the composition of structures and differences therein. QSM provides information on the magnetic susceptibility differences in the imaged tissue or region. As such it provides information on materials with varying magnetic susceptibility. $T_{2}^{*}$ provides information on the signal decay, that is affected by the magnetic susceptibility and the relaxation. Materials can have vastly different susceptibilities (i.e. paramagnetic, diamagnetic and ferromagnetic materials, all of which contribute to $T_{2}^{*}$ relaxation, but can be separated using QSM) dependent on their composition and constituents. These in turn distort the local magnetic field and thus affect signal localization and relaxation. The water-plant interface can pose problems when (paramagnetic) air is introduced on to the surface of structures or trapped within the dense root network causing effectively large dark and distorted areas in the MR images. Some of these local magnetic field distortions arising from air bubbles trapped within the roots seemed to extend over the pith structures as well. Nevertheless, quantitative susceptibility mapping could provide information on the differences in the crown structures between healthy and infected tissues. Other materials, such as nutrients may exhibit their own characteristic magnetic susceptibilities. As a clear paramagnetic region is observable in the center of the pith in the presented healthy example (Fig. 4), the lack of signal in MGE acquisition could be caused by magnetic susceptibility differences. This observable effect can be, for example, due to increased porosity (more paramagnetic air in the region), or due to accumulation of para- or even ferromagnetic nutrients.

In the mean proton density $S_{0}$ maps (Fig. 7), no significant intensity differences are observable in the pith between the healthy and inoculated examples. As such, the differences in the pith during the development are more clearly observed as relaxation time changes rather than changes in the concentration or distribution of the ^1^H nuclei (i.e. protons). For both healthy and infected samples, a lower $S_{0}$ signal is observable at the center of the pith region, and a higher $S_{0}$ signal near the crown-stolon junction, i.e. the interface located at the intersection of the stolons and the crown and from which the adventitious roots originate. However, a signal increase is observable in the healthy examples outside the pith near the leaves of the plants.

Interestingly, typical interpretation of the initiation of the *P. cactorum* infection is attributed, at least in some cases, to the exposed stolon interior (the “stolon stump”). However, in our work not all stolon cutoffs were submerged underwater and thus the exposure to the pathogen may have alternative routes, such as cuts and injuries (the stolons of healthy plants #2 and #3 as well as inoculated plant #4 (see Supplementary Fig. S1 online) were not submerged in water in the hydroponic system). As the relaxation time parameters exhibit spatial and temporal changes (as seen in Fig. 6) near the adventitious roots, these could be, at least in this case, a pathogen invasion route as well. For stolons, the increase in relaxation times during infection is not visible (Fig. 3, 4 and 7; see Supplementary Fig. S1 online), which may be due to the different pith cell composition between the stolon and the crown^5^. Moreover, the elongated stolon structures were not the emphasis of this work, rather, imaging was focused on the structures in the crown, namely the pith and vascular tissue structures. In the photographs of the final time point (week 2, see Supplementary Fig. S1 online) crown rot is observable in all inoculated plants. However, for a single inoculated plant (#4, see Supplementary Fig. S1 online) the effect of crown rot is not observable near the crown-stolon junction but near the opposite end of the pith. Even with such a plant sample, the relaxation time changes occur at or near the crown-stolon junction. The observed changes in the relaxation times may be caused by a plant’s response to the infection or halt in development rather than from the direct crown rot itself. Nevertheless, the propagating edge of the infection, observable in the photographs, aligns reasonably with the edge observable in the $T_{2}$ maps. Visible progression of *P. cactorum* infection was evident at the final time point as apparent darkening of the stolon and crown (Fig. 8).

Although the effects of *P. cactorum* are evident in the processed quantitative MR data, limitations, and practicalities regarding the use of MRI when examining plant physiology should be considered. First, the low sample size can be seen as problematic, although not atypical for MR experiments regarding method development^33^. Although the suitable sample size should be carefully considered as inter-sample variation is evident in strawberry plants, the observed difference is statistically significant in $T_{1}$, $T_{2}$ and $T_{2}^{*}$ maps between the two groups near the crown-stolon junction (0–50%) already on week 1 and even more so on week 2 as presented in Fig. 6. For $T_{2}^{*}$, statistically significant differences near the crown-stolon junction could be observed on week 0 as well. Inter-sample variation namely in the inoculated batch is observable (Fig. 5).

Another limitation is in the plant positioning for MRI. Although considered during the data acquisition, the MR imaging configuration and pre-handling resulted in slight variations in the slice selection and orientation. While this effect is minimal in the crown region itself, the surrounding narrower structures (e.g. stolon and roots) can be considerably affected. This effect is further amplified by the natural growth of the plants, making a precise and identical slice selection between the time points cumbersome, or even impossible. Another aspect to consider is the absolute numerical values obtained for the presented plant areas, and changes therein. Although comparable between plants in this study, the relaxation times are dependent on the magnetic field strength^34^ as well as other experimental parameters. Thus, although the absolute numerical values presented are comparable within a single study, i.e. with the same pulse sequence, imaging parameters, utilized RF coils and analysis as well as the same magnetic field strength, changes in these can affect the observed relaxation values.

Lastly, better temporal resolution, namely a daily MRI investigation, would be beneficial for the investigation of pathogen processes and for defining a more precise limit of detection. In the future, soil-based infection propagation could be investigated as well, although, for example, soil material with zero magnetic susceptibility should be used to avoid artifacts in the acquired data.

The purpose of this study was to investigate with magnetic resonance imaging (MRI) the changes and developmental differences caused by *Phytophthora cactorum* in the tissue of strawberry crowns. It was observed that quantitative parameters, specifically MR relaxation time values, provide information on the progression of crown rot as $T_{2}$ and $T_{2}^{*}$ values were significantly altered during *P. cactorum* infection. Similar changes were observed with $T_{1}$ relaxation times. Quantitative susceptibility mapping provided information on the magnetic susceptibility of different crown regions and may explain some of the differences between $T_{2}$ and $T_{2}^{*}$ values. This study shows the great potential for utilizing 2-D or 3-D quantitative MRI in longitudinal investigations on plant development, tissue damage and disease progression. The used imaging methods will help in the non-invasive monitoring of the temporal dynamics of pathological changes and thus, provide information that can be utilized to understand the pathogenesis of plant diseases and their progression.

## Materials and methods

### Strawberry plant samples

The *Fragaria* × *ananassa* plant material (cultivar Malling Centenary) was grown at the facilities of the Department of Environmental and Biological Sciences, at the University of Eastern Finland (*Kuopio, Finland*). The use of plants in the present study complies with international, national and/or institutional guidelines. Batches of inoculated ($N$ = 4) and healthy ($N$ = 4) *Fragaria* plantlets were grown in a hydroponic system as separate experiments. For both batches, the strawberry plantlets were placed in individual open-ended ⌀20 mm cylindrical acrylic tubes and grown in a hydroponic system. This system consisted of a plastic container (34 cm × 25 cm × 27 cm) covered with a lid, four acrylic tubes protruding through the lid, an aeration tube, and an air stone. The container interior was sealed from light to prevent algal growth. The container was filled with nutrient solution containing 0.5 g/l Ferticare 7-9-32 and 0.5 g/l YaraLiva Calcinit (Yara International ASA, *Oslo, Norway*) and solution was aerated with pressurized air. LED lights (LM12, FSG spectrum, Sunritek, *Shenzen, China*) were on for 16 h per day and the temperature was 20 ± 2 °C.

The plants were grown hydroponically for 10 days prior to the introduction of the pathogen. The zoospores of *P. cactorum* isolate KRJ1^35^ were produced as described earlier^36^. For the infected batch, inoculation was done by adding zoospores to the nutrient solution. After 1–2 days after the inoculation, the plants were MR imaged. For MRI, the nutrient solution from the hydroponic system was ejected from the acrylic tube and replaced with tepid tap water. The acrylic tube was sealed from the bottom and the leaves of the plant were folded loosely together with paper to protect them from mechanical damage and to minimize potential for contamination of the intact plants via the bore of the MRI device. After MR imaging, the tap water was evacuated and the plant together with the acrylic tube was returned to the hydroponic system (plants growing in the acrylic tubes visualized in Fig. 8). From the sample plants, the mother plant stolon was kept above water for one plant in the *Phytophthora cactroum* inoculated batch (designated #4), and two plants (#2 and #3) in the healthy batch (see Supplementary Fig. S1 online).

### Magnetic resonance imaging

MRI data were acquired at the facilities of the Kuopio Biomedical Imaging Unit, University of Eastern Finland, Kuopio, Finland (part of Biocenter Kuopio, Finnish Biomedical Imaging Node and EuroBioImaging). The MR imaging was accomplished with a 9.4 T vertical magnet (Varian/Agilent) and the accompanied software (VnmrJ 3.1.) utilizing a ⌀20 mm ^1^H RF coil (RAPID Biomedical GmbH, *Rimpar, Germany*). All MRI experiments were conducted at room temperature. Appropriate calibration, i.e. tune-matching of the RF coil, power calibration and shimming was performed prior to MR imaging. The healthy and inoculated plants were grown and imaged separately to avoid cross-contamination. During their development, the plants were MR imaged weekly to observe possible changes caused by *P. cactorum*, and more specifically, the progression of crown rot. The plants were imaged with an interval of 7 ± 1 days (once a week) for a total of two weeks (designated as week 0, week 1 and week 2).

The MR imaging parameters and the utilized pulse sequences are presented in Table 1. FSEMS-IR designates an implementation of an inversion recovery fast spin echo (multi slice), MEMS a multi spin echo (multi slice) and MGE a multi gradient echo pulse sequence used to obtain quantitative $T_{1}$, $T_{2}$ and $T_{2}^{*}$ maps, respectively. For FSEMS-IR and MEMS, identical image sizes, slice orientations and fields-of-view (FOVs) were used. The total scan times of the presented measurements amounted to approximately 1.5 h per experiment (Table 1).

**Table 1:** MR imaging parameters for each utilized pulse sequence.

Pulse sequence	Use	Mode	TR (ms)	TI/TE (ms)	Image size	FOV (mm)	Slice thickness (mm)	Slice gap (mm)	Scan time (min)
FSEMS-IR (fast spin echo multi-slice with inversion recovery)	$T_{1}$, structure	2-D	5000	10 23 51 115 260 588 1330 3000	512 × 256	36 × 18	1.0	0.5	21.5
MEMS (multi-echo multi-slice)	$T_{2}$, structure			TE_1_ = 10, ΔTE = 10, 10 echoes					23
MGE (multi gradient echo)	$T_{2}^{*}$, QSM, structure, crown volume	3-D	80	TE_1_ = 2.3, ΔTE = 4.1, 6 echoes	384 × 192 × 192	38.4 × 19.2 × 19.2	0.1	–	49

TR designates the repetition time, TI inversion time, TE echo time, FOV field-of-view, mode the imaging mode (slice-selective or two phase-encoding directions) and QSM quantitative susceptibility mapping. TE_1_ represents the shortest echo time and ΔTE the echo spacing. For FSEMS-IR the varied parameter is TI, and for MEMS and MGE the varied parameter is TE. The number of averages for each sequence was one.

### Plant dissection and reference photography

At the end of an entire experiment (i.e. after the MRI experiment on week 2), the plant was dissected and photographed to provide visual insight into the internal structures. The dissection was carefully matched with the acquired MRI slices as much as possible by dissecting the plant vertically in two equal halves equaling roughly to the middle slice obtained with the two spin echo sequences (MEMS, FSEMS-IR). This allowed the observation of the possible progression of *P. cactorum*, namely alterations caused by crown rot. Light photography setup was readied before dissection and photography was conducted immediately after the dissection to circumvent the effects of rapid oxidation in the exposed internal structures catalyzed by phenolases.

### Calculation of relaxation time maps

From the acquired FSEMS-IR, MEMS and MGE data, $T_{1}$, $T_{2}$ and $T_{2}^{*}$ relaxation maps were calculated, respectively. For FSEMS-IR, minimal de-noising via *k*-space filtering was performed. Parametric values were fitted with MATLAB (R2021b, MathWorks, Inc. *Natick, MA*) using fminsearch by solving for two parameters, proton density $S_{0}$ and suitable relaxation time, i.e. $T_{1}$, $T_{2}$ or $T_{2}^{*}$ for each image voxel. For relaxation time mapping, visualization, and ROI selection, an in-house built GUI for MATLAB was utilized (aedes, http://aedes.uef.fi/) with appropriate in-house written plugins and additional MATLAB functions and scripts.

For data obtained with the FSEMS-IR pulse sequence, proton density $S_{0}$ and $T_{1}$ maps were calculated pixel-wise using equation

$$S\left( t \right) = S_{0} \left( {1 - 2\exp \left( { - \frac{t}{{T_{1} }}} \right)} \right).$$

Conversely for MEMS and MGE pulse sequences, proton density $S_{0}$ and $T_{2}$/$T_{2}^{*}$ maps were calculated for each pixel or voxel using equation

$$S\left( t \right) = S_{0} \exp \left( { - \frac{t}{{T_{2}^{\left( * \right)} }}} \right),$$

where $T_{2}^{\left( * \right)}$ represents $T_{2}$ for MEMS data and $T_{2}^{*}$ for MGE data (Table 1). $T_{1}$ and $T_{2}$ maps were calculated for the entire image sets. From the proton density $S_{0}$ maps obtained together with $T_{1}$ and $T_{2}$ maps, arithmetic mean was calculated after min–max normalization.

For $T_{2}^{*}$ mapping, near-zero intensity values were excluded from the MGE data by using a threshold of 1.5 times the average signal intensity from the image set obtained with the shortest echo time ($t$ = TE_1_). For further analysis of the $T_{2}^{*}$ maps and to produce comparable information to other relaxation maps, arithmetic mean of 10 consecutive relaxation time map slices was calculated.

To illustrate the spatial dependence in the calculated relaxation times, profiles from the crown pith region were extracted. These were obtained from a narrow strip extending from the crown-stolon junction to the opposite side of the crown, i.e. near the vascular tissue near the leaves as illustrated in Fig. 1B. This was achieved by spline-interpolating two manually defined ROI curves between the abovementioned endpoints and sampling the relaxation time values over a width of 10 pixels along the path centered between these two manual ROI curves. To enable comparison between the plants, the lengths of the profiles were interpolated to the longest profile for each time point. From these, point-wise arithmetic means and standard deviations were calculated. The mean relaxation time profiles and their standard deviations were then presented as a function of a normalized distance from the crown-stolon junction (0%) to the vascular tissue at the opposite end of the pith of the crown (100%) (Fig. 1B).

### Calculation of quantitative susceptibility maps

Quantitative susceptibility maps (QSM) were calculated from the 3-D MGE data. Because QSM is based on the convolution of the entire image data, a region-of-interest (ROI) was generated, which omitted near-zero signal intensities from the image set obtained with the shortest echo time (TE_1_) using a threshold of 1.5 times the average signal intensity. Although minimal signal intensity was observed in some pith regions, they were included in QSM calculation. The regions outside the ROI were used as background signal for the QSM algorithm. QSM processing was done by using the MEDI toolbox for MATLAB^37^: complex fitting was done for the phase data, followed by Laplacian phase unwrapping and background field removal using projection onto dipole fields method (PDF). The susceptibility inversion was done with MEDI-L1, with a lambda value of 10,000.

### Estimation of crown volume

Crown volume estimate for each plant and time point was calculated from the three-dimensional MGE data set (shortest TE). As the crown pith volume is easily defined, the crown volume was selected as an appropriate region of interest (Fig. 1C). The volume was obtained by manually segmenting the approximate crown region, calculating the number of voxels and corresponding them to the MR data voxel-size (100 µm isotropic voxels). The crown volume was restricted to the pith regions, i.e. stolon and vascular tissue structures were omitted.

### Statistical evaluation

The groups of healthy and inoculated strawberry plants were investigated with two-sample *t*-test with MATLAB function ttest2. The statistical test was done for each discretized profile region illustrated in Fig. 1B between healthy and inoculated plants for each time point.

### Visualization of data

For the visualization of the measured 2-dimensional slice MR data, mask was applied for the $T_{1}$ and $T_{2}$ maps, effectively setting regions with excessive and null relaxation values to zero, i.e. signal from the water surrounding the plant and air outside the acrylic tube, respectively. Similar procedure was conducted for the presented $T_{2}^{*}$ maps. Furthermore for $T_{2}^{*}$ and QSM maps, mean intensity values for 10 consecutive slices were calculated. This amounted to the same slice thickness as for the 2-dimensional data ($T_{1}$ and $T_{2}$ maps).

Three-dimensional visualizations were obtained from the $T_{2}^{*}$ maps calculated from the measured MGE data for an inoculated and healthy strawberry plant at the three time points. This was achieved by exporting the relaxation data set into a suitable file format (DICOM) using MATLAB and rendering it volumetrically in pseudo-color using Horos image viewer for MacOS (https://www.horosproject.org/). 3-D volume renderings of a single plant were compared to photographs taken each week before MRI.

### Supplementary Information

Supplementary Information 1.

Supplementary Video 1.

## Data Availability

Data, including the measured MR data and calculated relaxation time maps, are made openly and fully available at the Zenodo repository Doi: (10.5281/zenodo.7442381). All custom MATLAB scripts are available from the corresponding author upon a reasonable request.
