# Supplementary material for: Non-invasive assessment and visualization of Phytophthora cactorum infection in strawberry crowns using quantitative magnetic resonance imaging
Source: Sci Rep. 2024 Jan 25;14:2129. doi: 10.1038/s41598-024-52520-7 (PMC10808117; doi:10.1038/s41598-024-52520-7)
Supplement: Supplementary file 1 — Supplementary Information 1. [file 41598_2024_52520_MOESM1_ESM.pdf]

Supplementary information:

Non-invasive assessment and visualization of Phytophthora cactorum infection in strawberry crowns using quantitative magnetic resonance imaging

Teemu Valtteri Tuomainen[1]; Anna Toljamo[2]; Harri Kokko[2]; Mikko Johannes Nissi[1,]*

[1] University of Eastern Finland, Department of Technical Physics, Yliopistonranta 8, FI-70210, Kuopio, Finland

[2] University of Eastern Finland, Department of Environmental and Biological Sciences, Yliopistonranta 8, FI-70210, Kuopio, Finland

Supplementary data

Figure S1. Example of T_2 maps and corresponding photographs.

Video S1. Rotating 3-D pseudo-color volume renderings of healthy and inoculated plants at different time points.

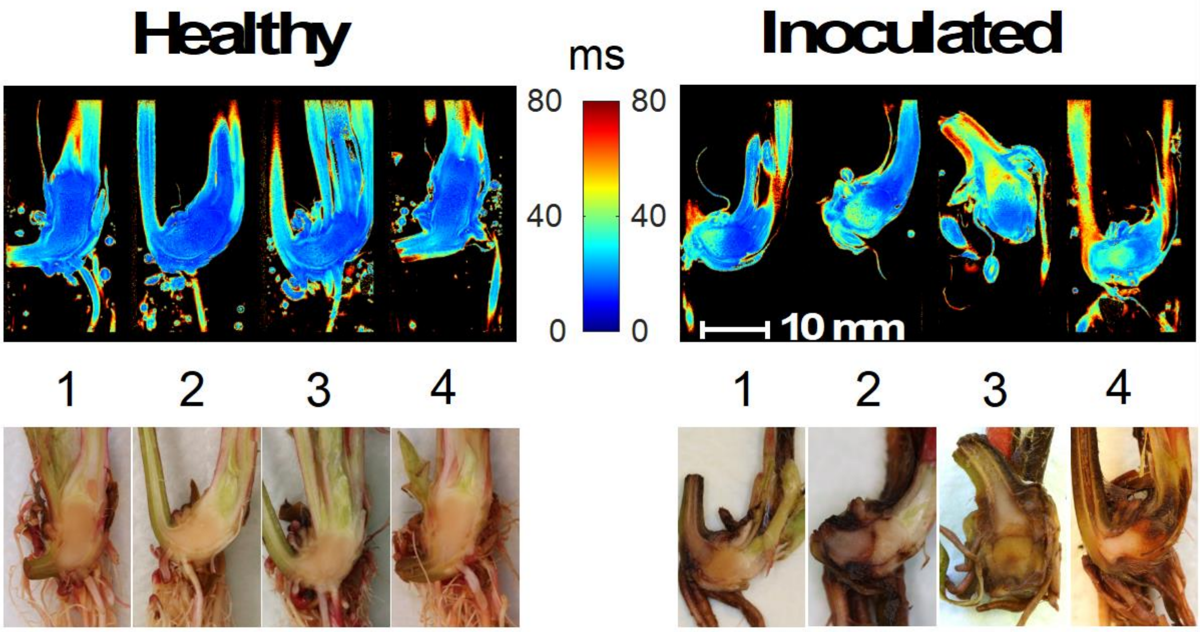

Fig. S1. Example of T_2 maps and corresponding photographs. T_2 relaxation time maps of the healthy and inoculated strawberry plantlets on week 2 and the corresponding photographs of the dissected plantlets. For clarity, T_2 values from surrounding water have been omitted. Please note the difference in the stump lengths, deliberately cut as shown to assess the effect of the infection route. The color gradient represents the relaxation times for each region, from relatively low relaxation time values (blue) to relatively high relaxation time values (red), and relaxation time values in-between (cyan, green, yellow and orange).
